# A Golf Course Quality Scale (GCQS): Validity and Reliability Testing on Spanish Golf Courses

**DOI:** 10.3390/ijerph182413301

**Published:** 2021-12-17

**Authors:** Francisco Cavas-García, Alfonso Martínez-Moreno, José María López-Gullón, Arturo Díaz-Suárez

**Affiliations:** Department of Physical Activity and Sports, CEIR Campus Mare Nostrum (CMN), University of Murcia, Santiago de la Ribera, 30720 San Javier, Spain; luchamurcia@um.es (J.M.L.-G.); ardiaz@um.es (A.D.-S.)

**Keywords:** perceived quality, validity, reliability, golf courses

## Abstract

The objective of this study was to evaluate the psychometric properties, in terms of validity and reproducibility, of the Golf Course Quality Scale (GCQS) in the Spanish golf course context. The GCQS is a scale that measures the quality of a golf course perceived by its users. It is comprised of 22 five-point Likert scale items, ranging from 1 (disagree) to 5 (totally agree). The items were grouped into five dimensions: services, etiquette, facilities, golf courses, and staff. A translated and adapted version of the Australian CQS questionnaire was administered to a total of 401 federated golf players in the Region of Murcia (RM). The margin of error was 4.9% with a confidence level of 95%. Of the total sample, 88.5% were men and 11.5% were women. The participants were between the ages of 18 and 80 (*M* = 54.1, *SD* = 14.3). Following an exploratory factor analysis (EFA) to determine the suitability of the proposed items and the factorial structure of the scale, a confirmatory factor analysis (CFA) was performed using structural equations under the maximum likelihood extraction method. This assessed the fit of the model and its internal consistency, with values above 0.79. In conclusion, the validity and reliability of this psychometric scale for the purposes of evaluating the quality of golf courses perceived by their users have been verified. The results confirm that the scale is a useful tool for golf course research and management.

## 1. Introduction

The sports sector in Spain has evolved significantly, adapting to the social and economic context to respond to the high-quality standards demanded, while generating a series of adaptations and a wide range of services responding to the current demands of sport organizations. Méndez-Rial [1] suggested that management teams should have flexible organizations, with well-trained professionals and new management, as well as management systems, for the promotion of research in sports services.

In the field of individual sports, golf, alongside tennis, is undoubtedly one of the most popular sports in terms of participation and follow-up of professional events [2]. The growing concern for health in society means that the golf sector within the sports economy must adapt to modern times, evolving from a traditionally elitist sport to one that could adapt to demands created by society today. To this end, principles of management must be understood globally and accompanied by high-level professional training to allow for understanding and adapting to the future of the golf market.

### 1.1. Implementation of the Golf

The importance of golf as an activity that generates resources is not limited exclusively to the income generated by the practice of the game. There are numerous considerations to make when offering a global vision of the golf market, also highlighting its close relationship with other sectors [2].

According to data from the official R&A report, Golf Around the World [3], at the end of 2018, there were 38,864 golf courses distributed across the world, most of which were concentrated in Western countries: the United States, Japan, Canada, England, Australia, Germany, France, the Republic of Korea, Sweden, and Scotland. In relation to Europe, Spain ranked sixth in the world, with a total of 305,417 federated players, close to the top 5 European countries, led by Germany with 642,240 followed closely by England with 629,000 [4]. According to the Royal Spanish Golf Federation (RSGF) and the Spanish Association of Golf Courses (SAGC) in their latest report on the golf sector [5], the golf sector in Spain attracts a total of 1,195,000 foreign tourists, 98.6% of whom come from the European continent, making Spain the leading country in golf tourism in Europe. This, in turn, directly and indirectly generates 121,395 jobs.

The Region of Murcia (RM) has a total of 21 golf courses, mainly located in the surroundings of the region’s Mediterranean slope. The RM is a Spanish autonomous community that has registered the highest growth in terms of golf equipment throughout the national territory and has grown from having 4 golf courses in 2003 to 21 today. Consolidating great tourism and sports for the RM, attracting high-level athletes and with high purchasing power, this has an impact on the regional economy as it allows the organization of international sporting events. According to the latest balance of the Tourism Institute of the Region of Murcia in 2018 [6], the RM received a total of 152,000 golfers who generated the acquisition of 520,840 green fees.

### 1.2. Quality of Service

The concept of quality of service in the field of golf equipment management is considered one of the most key issues for company performance, as it is an element of vital importance to the survival and advancement of organizations. Any service or product offered by a company must answer to the demands of the client, as well as possess sufficient characteristics to respond to customer expectations to be valued positively by the client.

Although the definitions of quality in the literature have varied, there are two theoretical models, the Nordic School and the American School. These models, or schools, are the most representative in terms of the study of the perceived quality of services [7]. The Nordic model is mainly based on Grönroos [8] and the North American model is based on Parasuraman’s [9,10] research. To measure and quantify the concept of quality of service, the SERVQUAL and SERVPERF scales are two psychometric scales that have had a greater diffusion throughout recent times. Fidelity is a determining factor, to ensure that organizations endure over time [11], as well as where to focus your efforts in terms of satisfying customers to turn them into loyal customers [12], which attributes are fundamental to the perception of a golf course, and which are of lesser importance to customers [13].

Recently, [14] analyzed the existing scales and instruments measuring quality for the sports services sector. Ramírez et al. [14] indicated that while most of the scales used are based on the SERVQUAL and SERVPREF, new scales have also been developed in recent years, for example, Q-GOLF9 [15], while in the Spanish golf sector, there are not many specific tools compared to other more generalist sports facilities. Scales in this sense have been used in other countries, so we used a translated and adapted version of the CSQ [15] used in Australian golf courses in order to provide a valid and usable tool for the Spanish context.

These tools have followed guidelines stating that any instrument for measuring the quality of future services should include assessments that study the following aspects: (i) the facilities and their state of accessibility, cleanliness, and maintenance, whether sports facilities or complementary; (ii) the equipment and materials available at the facility; (iii) the environment; (iv) the scheduling of activities and features of this scheduling; (v) the attention paid to the client and communication; (vi) the safety and reliability of the facilities; (vii) the pricing of the service with respect to the market and location; (viii) the image of the organization; and (ix) the personal relationships between customers, as well as customers and employees, inside and outside of the premises.

Previous research has suggested that service quality is influenced by both core elements (main functions) and peripheral elements (secondary services) [16]. The elements to consider in golf with respect to other sports, apart from the service, the facilities, and the staff, are the etiquette and the golf course, both differentiating elements of this sport. Some golf clubs remind players of the required attire on the course and in the clubhouse, and even print the dress code on the green fee ticket [17]. Commentators and players often state that “etiquette is arguably more important in golf than in any other game” [18]. The little academic research that has focused on golf etiquette suggests that it has considerable exclusionary rather than inclusionary elements [19].

Knowledge of the needs and expectations of the users allows organizations to implement strategies that reach users’ present expectations while simultaneously predicting future ones. This, in turn, generates greater long-term user loyalty. With the right tools for studying the quality perceived by users on golf courses, it would be possible to contrast the different strategies implemented by managers to improve the services offered at their courses and the impact of these strategies on user satisfaction. Golf courses, as with other sports facilities, need tools that help to properly evaluate the quality perceived by the user. This need is explicitly reflected in the Spanish standard, UNE:188001, on golf course requirements for the provision of services [20].

In response to the increasing sensitivity of golf players to the need for a sustainable quality service that includes environmental measures [21], there are other standards in Spain regarding the environmental management of golf courses, such as ISO:14001, which contemplates a model of an environmental management system whose objective is the control of production methods and the provision of environmentally friendly services [22].

All this together with the fact that we do not currently have a large number of tools available in the field of golf for this purpose, taking into account that these must have criteria of validity and reliability, led us to carry out this study. Coupled with the importance of the golf sector, both nationally and in the RM, this research aimed to verify the validity and reliability of a psychometric scale to evaluate the quality perceived by the users of golf courses in the context of golfing in Spain.

## 2. Materials and Methods

The research used a quantitative, transversal, and observational design to understand the needs and expectations of golf course users in relation to the elements of perceived quality of golf courses.

The study was carried out within the framework of the Declaration of Helsinki [23] and with the favorable report of the Research Ethics Commission of the University of Murcia nº ID: 2491/2019.

### 2.1. Participants

The present study contemplated a theoretical universe constituted by all users with a federative license, which was 5.420 according to the RSGF count at the time of administration of the questionnaire (i.e., July 2017). To calculate the representative sample size for this universe, the following formula was applied for finite populations:

$$n=\frac{N}{1+\frac{e^{2}\left(N-1\right)}{z^{2}pq}}$$

where:

*n* = sample size (to be determined)

*N* = population size = 5.420

*e* = sampling error = 0.05

*z* = *z*-value corresponding to the confidence level

*pq* = population variance

The result of the above calculation determined that with a maximum allowed margin of error of 5% for a confidence level of 95%, and under the assumption of maximum variance (*p* = *q* = 0.5), a sample size of 359 users was necessary. A final sample of 401 users was obtained with a margin of error of 4.9% for a confidence level of 95%.

### 2.2. Procedure

The procedure used to carry out the research comprised six stages. First, the Golf Federation of the Region of Murcia (GFRM) was contacted, and the objective of the study was explained to obtain their support. Next, information was sent to federated users, and information was sent to all federated users in July 2017. Simultaneously, a link to the questionnaire in both Spanish and English was published on the federation’s website and a statement was sent to the local media for dissemination. The questionnaire was administered electronically between July 2017 and June 2018 and was accessible both on the official GFRM website and through the corporate email sent at the beginning of the study. Once the data collection period had ended and an acceptable number of responses, according to the sample size estimate, had been obtained, a data dump was performed to carry out statistical treatment.

### 2.3. Instrument

For the purposes of the present study, a translated and adapted version of the CQS questionnaire [24], which had originally been developed to measure the perceived quality of Australian golf courses, was used. The scale for perceived quality was adapted and used for the analysis of the quality of golf courses in Andalusia [25]. The new scale renamed GCQS (Golf Course Quality Scale), as shown in Table 1, is a psychometric questionnaire comprising 22 Likert scale items, with values ranging from 1 (disagree) to 5 (totally agree). These items have been grouped into 5 dimensions: services (Q1, Q2, Q3, Q8, Q9, and Q21); etiquette (Q5 and Q10); facilities (Q5, Q6, Q7, and Q19); golf course (Q11, Q12, Q13, Q14, Q20, and Q22); and staff (Q15, Q16, Q17, and Q18). This scale collects information on the quality of a golf course as perceived by the user of the golf course and focuses on each of the most relevant aspects that serve as indicators for the management of these facilities.

### 2.4. Statistical Analysis

For the statistical analysis of the results, IBM’s SPSS (Statistical Package for the Social Sciences) and AMOS, both version 24.0, were used. A significance level of *p* < 0.05 was assigned to all tests.

For construct validation of the quality scale, an EFA was first performed to determine the suitability of the proposed items, as well as the factorial structure of the questionnaire, using the main component method with a Varimax rotation. Next, a CFA was performed after a model was generated in the exploratory study. This was carried out with the use of structural equations, using the method of extraction of maximum likelihood. The fit of the model was assessed using the following fit indices: the goodness-of-fit index (GFI), adjusted goodness-of-fit index (AGFI), comparative fit index (CFI), normed fit index (NFI), Tucker Lewis index (TLI), and root-mean-square error of approximation (RMSEA). Internal consistency, measured using Cronbach’s alpha, was also confirmed.

## 3. Results

In this section, the results of the data analysis are explained. There were 401 total users who formed the final sample of the study. These were users of the golf courses of the MR. Of the total, 88.5% were men and 11.5% were women, aged between 18 and 80 years (*M* = 54.1, *SD* = 14.3). As for nationality, 56.1% were Spanish and 43.9% were foreign nationals, 62.9% of which were of English nationality.

### 3.1. Exploratory Factor Analysis

The EFA was developed following the main components with Varimax rotation (see Table 2). Five factors with eigenvalues greater than 1 were observed, following a criterion to place an item to each factor whose factorial load was above 0.3, explaining, among the five factors, 65.9% of the total variance. The correlation matrix showed an outstanding number of correlations (87.7%) that had obtained a value over 0.3, with a determinant equal to 2.42 × 10–6. The result of the Bartlett sphericity test determined the nonindependence of the variables (Bartlett Test = 5066.73 (g.l. = 231), *p* < 0.001). Additionally, the Kaiser–Meyer Olkin (KMO) test, which seeks to show the adequacy of the sample, was 0.946 and the commonalities were higher than 0.61. Finally, the set of values of the measures of the sampling adequacy (MSA) test stood above 0.85. In summary, the values outlined offer viability to the factor analysis of the correlation matrix.

### 3.2. Confirmatory Factor Analysis

**Figure 1:**
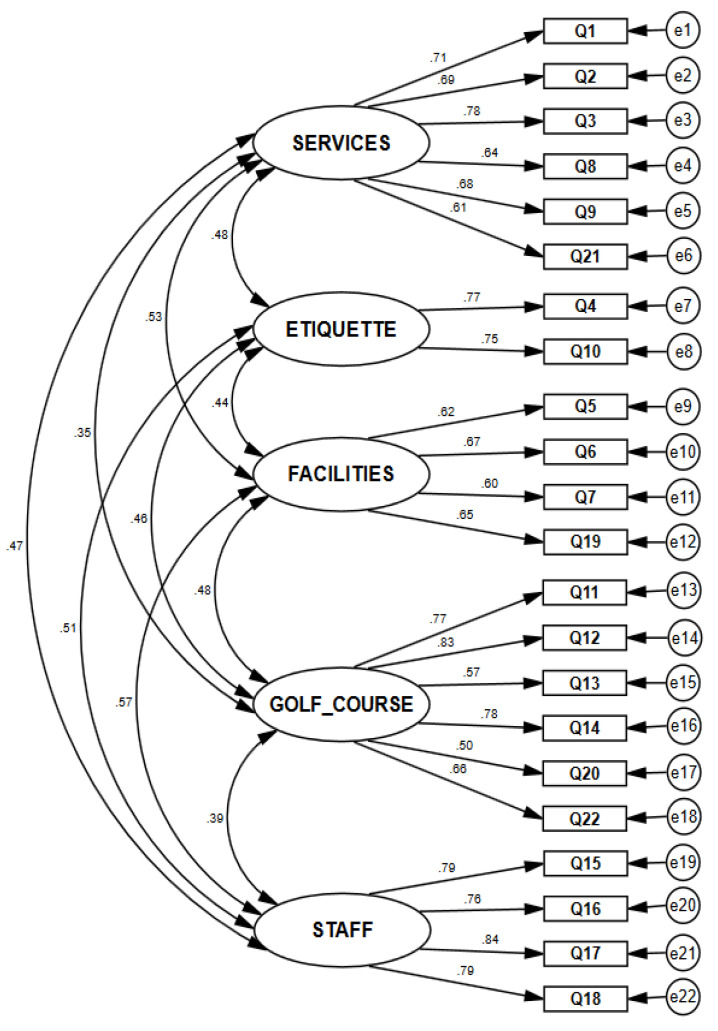
Confirmatory factor analysis.

After the aforementioned model had been generated in the EFA, the CFA (see Figure 1) was performed with structural equations using the maximum likelihood extraction method.

The adequacy of the model obtained in the EFA was confirmed, as a model composed of five factors and 22 indicators in total was obtained. The estimated parameters were statistically significant (*p* < 0.05), and the factorial loads presented values higher than 0.5 such that all of the indicators saturated satisfactorily with each of their latent variables, and the values of the average variance extracted (AVE) were greater than 0.60, suggesting convergent validity. Regarding internal consistency, as demonstrated in Table 3, Cronbach’s alpha and composite reliability values were above 0.75. The discriminant validity was also adjusted in an acceptable way, as in all cases, the square root of the AVE of each of the constructs was greater than the correlation of one with any other.

In relation to the adjustment of the model (see Table 4), the various adjustment indices were adequate. Thus, the proposed model of the factorial structure of the scale was sustainable. In addition, in the analyses carried out on two random subsamples, the results were also satisfactory.

## 4. Discussion

The concept of quality of service encompasses not only customer satisfaction, but also the repetition and use of the service, along with subsequent recommendations to other customers. Therefore, much like any other business organization, sports, or sports-tourism facilities (e.g., golf courses), must be aware of the needs and desires of users to implement strategies aiming to meet the current and future expectations of users. To do this, managers require tools that offer tangible results with the goal of generating greater user loyalty and loyalty in the golf courses they manage.

Ramirez et al. [14] indicated that, over the past few years, in the field of sports management, studies have been carried out with the aim of creating instruments that measure the quality of sports services, e.g., Chulhwam et al. [26], in virtual golf

This fact has an impact on the fact that the measurement of the quality perceived by the user of sports equipment is fundamental for the successful management of sports facilities [27].

Examples of scales adapted from the SERVQUAL model according to the services offered, used in quality measurement, are as follows [28,29,30,31,32,33,34], whose main characteristic is the difference between the customer’s expectations before using the service and the actual service provided by the organization.

Following the analysis of the scale for perceived golf course quality, GCQS, based on the translated version and tested for content validity [24], we have verified that, after carrying out the EFA and CFA, the results allow us to retain the 5 factors and their 22 items contained in the initial scale. This confirms the discriminant validity, reliability, and high internal consistency with Cronbach’s alpha values above 0.8 in each of the five dimensions. In the golf sector, similar studies [15] have been found, although with other scales (QGOLF-9), which obtain values similar to those of this study.

These tools make it possible to operate with sufficient criteria of validity and reliability to determine which user perceptions affect the quality of the service offered. The customer is the one who values and provides essential information on the quality of the service provided by the organization.

In the present study, the scale for the perceived quality in the GCQS golf courses yielded results that allowed for the verification of the validity and reliability of this psychometric scale for evaluating the users’ perceptions of quality of the golf courses. Thus, the GCQS is a reliable and valid measuring instrument and a useful tool for research and management. It can be used by managers of the type of golf sports facilities featured in this research.

## 5. Conclusions

The main objective of the study was to verify the validity and reliability of the GCQS psychometric scale for the perceived quality of golf courses in the Spanish context.

The results obtained in this work have verified the validity and reliability of this psychometric scale for evaluating golf course quality as perceived by users. It has thus been concluded that the GCQS scale is a useful tool for golf course research and management. Its use by managers of golf facilities and golf courses in Spain would be advisable, providing valuable information to make decisions with objective criteria.

The results obtained after the application of the GCQS will facilitate decision-making by managers on aspects that directly affect the perceived quality of their users, such as complementary and additional services to the game, cleaning and maintenance of the equipment as a whole, and also on the design of the golf course itself. In this sense, the management of the golf equipment will obtain quality standards that will aim to obtain greater satisfaction and loyalty from the golf course customers.

As for the limitations of the present study, it would have been desirable to increase the sample size to extend the study to other geographical contexts and the problems encountered for data collection as it is a cross-sectional study and not exempt from problems, due to the situation of each golf course. As future lines of research, the incorporation to these types of scales of some items would allow one to know the opinion of the users of the golf courses about the care and sensitivity to the environment in the facilities and its repercussion in the perceived quality and satisfaction. It would also be possible to study the differences in perceived quality between golf courses with integrated residential housing vs. golf courses without residential housing.

## Figures and Tables

**Table 1:** GCQS scale items.

DIMENSIONS	ITEMS (22)—PERCEIVED QUALITY SCALE
**SERVICES**	Q1. Parking must be secure. Q2. Parking should be adequate. Q3. The facilities integrated in the golf course must be clean and tidy (shop, lounge). Q8. The pro-shop should have a wide range of golf equipment and accessories. Q9. The equipment in the pro-shop should be of high quality (carts and clubs). Q21.The website provides golf course information of ALL services provided.
**ETIQUETTE**	Q4. Information on golf rules, location, opening hours, etc. must be provided. Q10. Club rules and rules of etiquette must be respected.
**FACILITIES**	Q5. The course should offer various practice facilities for players. Q6. The course should offer competitions, facilities and programmes that provide value for money. Q7. The golf course must offer catering services (food and beverages). Q19. The golf course must have a social area
**GOLF COURSE**	Q11. The golf course must be of high quality and include appropriate elements (design, landscape, obstacles and field items). Q12. All areas of the golf course must be well maintained (tee, greens, bunkers). Q13. There must be sufficient water sources in the field. Q14. Adequate supply of support equipment (rakes, sand buckets) should be available on the golf course. Q20. All areas of the golf course must be prepared for people with reduced mobility. Q22. The condition of the greens is always in perfect condition.
**STAFF**	Q15. Golf course staff must be experienced and qualified. Q16. Golf course staff should be friendly. Q17. Golf course staff must be sensitive to the needs of customers. Q18. Golf course personnel should be presentable and easily identifiable.

**Table 2:** Exploratory factor analysis.

	Communalities	Factor				
		BE	TSI	FAC	GCO	STA
**Q1**	0.776	0.781				
**Q2**	0.794	0.823				
**Q3**	0.652	0.572				
**Q8**	0.774	0.799				
**Q9**	0.801	0.777				
**Q21**	0.621	0.514				
**Q4**	0.620		0.574			
**Q10**	0.644		0.686			
**Q5**	0.606			0.434		
**Q6**	0.488			0.480		
**Q7**	0.614			0.629		
**Q19**	0.682			0.704		
**Q11**	0.693				0.702	
**Q12**	0.773				0.767	
**Q13**	0.607				0.515	
**Q14**	0.643				0.598	
**Q20**	0.589				0.587	
**Q22**	0.613				0.659	
**Q15**	0.664					0.612
**Q16**	0.740					0.809
**Q17**	0.743					0.712
**Q18**	0.718					0.725
**Eigenvalues**		10.35	3.19	2.08	1.60	1.09
**% Variance explained**		16.36	16.11	12.42	10.70	10.34
**% Cumulative explained variance**		16.36	32.47	44.89	55.59	65.93

SER: Services, ETI: Etiquette, FAC: Facilities, GCO: Golf course, STA: Staff.

**Table 3:** Internal consistency, convergent validity, and discriminant validity.

	*α*	*ρ*c	AVE	SER	GCO	STA	FAC	ETI
Services	0.84	0.84	0.67	0.82 *				
Golf course	0.85	0.85	0.60	0.35 **	**0.77**			
Staff	0.89	0.88	0.64	0.47	0.39	**0.80**		
Facilities	0.86	0.77	0.66	0.53	0.48	0.57	**0.81**	
Etiquette	0.79	0.78	0.66	0.48	0.46	0.51	0.44	**0.81**

*α*: Cronbach’s Alpha; *ρ*c: composite reliability; AVE: average variance extracted; SER: Services, ETI: Etiquette, FAC: Facilities, GCO: Golf course, STA: Staff; * square root of the AVE on the diagonal; ** correlations between constructs below the diagonal.

**Table 4:** AFC goodness-of-fit indices.

	χ2 (g.l.)	*p*	χ2/g.l.	GFI	AGFI	CFI	NFI	TLI	RMSEA (I.C. 90%)
**Total**	221.78 (199)	0.128	1.11	0.96	0.95	0.96	0.94	0.97	0.054 (0.04–0.10)
**Subsample 1**	207.69 (199)	0.321	1.04	0.95	0.94	0.97	0.93	0.94	0.058 (0.04–0.11)
**Subsample 2**	199.34 (199)	0.479	1.00	0.96	0.95	0.95	0.95	0.95	0.056 (0.04–0.11)

Goodness-of-fit index (GFI); adjusted goodness-of-fit index (AGFI); comparative fit index (CFI); normed fit index (NFI); Tucker Lewis index (TLI); and root-mean-square error of approximation (RMSEA).
